# Evaluation of multisystemic therapy pilot services in Services for Teens Engaging in Problem Sexual Behaviour (STEPS-B): study protocol for a randomized controlled trial

**DOI:** 10.1186/s13063-015-1017-2

**Published:** 2015-11-02

**Authors:** Peter Fonagy, Stephen Butler, Geoffrey Baruch, Sarah Byford, Michael C. Seto, James Wason, Charles Wells, Jessie Greisbach, Rachel Ellison, Elizabeth Simes

**Affiliations:** Research Department of Clinical, Educational and Health Psychology, University College London, London, UK; Brandon Centre, London, UK; Centre for the Economics of Mental Health, Institute of Psychiatry, King’s College London, London, UK; Royal Ottawa Health Care Group, Ottawa, Ontario Canada; MRC Biostatistics Unit, University of Cambridge, Cambridge, UK

**Keywords:** Adolescent, Problematic sexual behaviour, Family, Multisystemic therapy, Randomized controlled trial, United Kingdom, Youth

## Abstract

**Background:**

Clinically effective and cost-effective methods for managing problematic sexual behaviour in adolescents are urgently needed. Adolescents who show problematic sexual behaviour have a range of negative psychosocial outcomes, and they and their parents can experience stigma, hostility and rejection from their community. Multisystemic therapy (MST) shows some evidence for helping to reduce adolescent sexual reoffending and is one of the few promising interventions available to young people who show problematic sexual behaviour. This paper describes the protocol for Services for Teens Engaging in Problem Sexual Behaviour (STEPS-B), a feasibility trial of MST for problem sexual behaviour (MST-PSB) in antisocial adolescents at high risk of out-of-home placement due to problematic sexual behaviour.

**Methods/Design:**

Eighty participants and their families recruited from five London boroughs will be randomized to MST-PSB or management as usual with follow-up to 20 months post-randomization. The primary outcome is out-of-home placement at 20 months. Secondary outcomes include sexual and non-sexual offending rates and antisocial behaviours, participant well-being, educational outcomes and total service and criminal justice sector costs. Feasibility outcomes include mapping the clinical service pathways needed to recruit adolescents displaying problematic sexual behaviour, acceptability of a randomized controlled trial to the key systems involved in managing these adolescents, and acceptability of the research protocol to young people and their families. Data will be gathered from police computer records, the National Pupil Database and interviews and self-report measures administered to adolescents and parents and will be analysed on an intention-to-treat basis.

**Discussion:**

The STEPS-B feasibility trial aims to inform policymakers, commissioners of services and professionals about the potential for implementing MST-PSB as an intervention for adolescents showing problem sexual behaviour. Should MST-PSB show potential, STEPS-B will determine what would be necessary to implement the programme more fully and at a scale that would warrant a full trial.

**Trial registration:**

ISRCTN28441235 (registered 25 January 2012).

## Background

Systematic reviews of the prevalence of sexual abuse in young people indicate that 18 to 20 % of females and 6 to 8 % of males in the general population report having been abused before the age of 18 [1, 2]. Adolescents are the perpetrators of approximately 20 % of all sexual offences, and victim reports suggest that the proportion of juvenile offenders (versus adult offenders) increases as the age of the victim decreases [3]. Victims of sexual assault are at a high risk for negative sequelae [4, 5], and sexual offenders themselves engender significant social and fiscal costs, including removal from their family homes and impaired social, academic and vocational competencies as a result of their sexual offending.

Sexual offenders are heterogeneous [6, 7] and include a substantive subgroup who show many of the same risk factors as juveniles who engage in serious antisocial behaviour that does not include sexual offending [8]. For example, several studies have shown that sexual offenders share many of the same family and peer risk factors seen in non-sexual offending youth such as poor parent supervision, lower parental involvement and poorer parent-adolescent relationships, lower bonding to family and school, and higher involvement with delinquent peers [8, 9]. At the same time, a recent meta-analysis concluded that adolescent sexual offenders, when compared with adolescent non-sexual offenders, show greater atypical sexual interests (for example, sexual arousal to children or to coercive sex) and histories of child sexual abuse, higher levels of anxiety and lower self-esteem, and greater social isolation [10]. Correspondingly, while the sexual reconviction rates for adolescent sex offenders range from 5 to 14 % and are substantially lower than their reconviction rates for non-sexual offences (16 to 54 %) [11–13], reoffending rates for sexual offending are often associated with sex-specific factors [13–16], while non-sexual recidivism is related to factors commonly predictive of general delinquency [13, 15].

Despite the evolving knowledge concerning juvenile sex offenders, much less progress has been made in developing effective interventions. In a recent systematic review of interventions designed to prevent reoffending among known sex offenders and individuals at risk of sexually abusing children, Langstrom and colleagues [17] were able to identify only eight intervention studies that fulfilled their criteria, including five prospective observational studies and three randomized controlled trials (RCTs); none of these studies had been conducted in the United Kingdom. The authors concluded that an implementation of multisystemic therapy (MST) tailored towards problematic sexual behaviours (MST-PSB) [18] showed limited evidence for helping to reduce sexual reoffending in adolescent sexual offenders. However, in a meta-analysis of adolescent and adult treatment programmes of sexual offenders, Hanson and colleagues [19] pointed to the benefits of MST-PSB for reducing adolescent problem sexual behaviour and highlighted MST as a rare example of an intervention that is consistent with the risk, need and responsivity principles for effective offender rehabilitation [20]. Importantly, one of the lessons learned from the adult treatment of sexual offenders is that adult sexual offenders who attend and cooperate with treatment programmes are less likely to reoffend than those who reject the interventions [21].

### Multisystemic therapy as an intervention for problem sexual behaviours

MST is an intervention that has been developed in the United States. It has an established track record for successfully treating young people who show serious and persistent antisocial behaviour [22]. More recently, MST has been transported to other countries with some promising (Norway [23–25]) and mixed (Sweden [26] and Canada [27]) results. In the United Kingdom, following an earlier single-site trial of MST [25], a large multi-site trial is now underway, which is comparing MST with the usual services delivered to this population of young people (the START trial) [28].

Multisystemic Therapy for Youth with Problem Sexual Behaviours (MST-PSB) is an adaptation of MST aimed at adolescents who have committed sexual offences and demonstrated other problem behaviours. It is an intensive family- and home-based intervention uniquely developed to address the multiple determinants of problematic sexual behaviour in adolescents. MST-PSB is designed to reduce problematic sexual behaviours, antisocial behaviours and out-of-home placements. Problematic sexual behaviour in adolescents is sexual behaviour that is developmentally inappropriate or potentially harmful to the self or others and includes behaviour of a sexual nature that infringes on the rights of others (such as the use of coercion or force) [29].

Recent reviews of the sexual offending outcome literature [17, 30, 31] have noted that two RCTs of MST-PSB are the only published and peer-reviewed RCTs to evaluate the effects of the intervention on juvenile sexual offending, and that they have produced promising findings [18, 32]. A small efficacy trial conducted by the developers of MST-PSB reported that juvenile sex offenders receiving MST were significantly less likely than those receiving usual services to be rearrested for either sexual offences (8 % versus 46 %) or nonsexual offences (29 % versus 58 %) at 8.9 years post-treatment, and to have spent 80 % fewer days in confinement facilities [18]. A larger effectiveness trial studying the impact of MST-PSB compared with usual services through a 12-month follow-up period reported that adolescents receiving MST-PSB engaged in less problem sexual behaviour and were less likely to be placed out of the home [32].

MST-PSB is therefore the only evidence-based intervention currently available for adolescents showing problem sexual behaviour. However, despite some initial positive findings, there are several unresolved issues relating to transporting MST-PSB to other health and social care systems and jurisdictions outside the United States. First, the effectiveness of MST-PSB needs to be carefully assessed in the UK mental health, juvenile justice and social care systems. The pattern of results found in the transportability of RCT evaluations of standard MST in Canada and Europe suggest that the effectiveness of MST-PSB needs to be demonstrated by independent investigators and outside the United States. Specifically, the magnitude of the associations between standard MST and treatment outcomes are substantially higher in trials that involved the developers of the intervention (effect size = 0.81) than in studies conducted without their close involvement (effect size = 0.27) [33], suggesting a possible ‘developer effect’ (for example, therapists supervised by developers adhere more to the MST guidelines or are more motivated and engaged). Thus, an independent transportability trial is necessary to demonstrate that MST-PSB can be delivered with fidelity by UK-trained therapists. This is crucial, given that the MST-PSB adaptation requires additional skills and practices that are not part of standard MST, such as addressing parents’ and adolescents’ denial of problematic sexual behaviour. Furthermore, the intervention is being applied by MST therapists to a highly stigmatized population against which there are very strong cultural biases and prejudices. The clinical competency of UK psychologists and social workers to deliver MST-PSB with fidelity, relative to their counterparts in the United States, may also be influenced by curriculum and training differences. In addition, the relative success of standard MST may be due to the relative quality of the usual services for managing antisocial behaviour in the United States compared with usual services in other countries. MST may produce better outcomes only when usual services produce weak, null or even negative effects. Thus, the superiority and cost-effectiveness of MST-PSB needs to be demonstrated outside the United States, in studies where the therapists delivering MST are independent of the MST-PSB developers, where the comparison services or ‘management as usual’ (MAU) is consistent with the options currently available for young people showing problem sexual behaviour in that region, and where the sentencing policy within the justice system does not result in comparison with alternatives such as custodial sentences. Moreover, as noted by Littell [34] following a detailed Cochrane review of the effectiveness of MST [35], it is crucial that the research evaluation team and the developers who deliver the clinical service be completely independent.

### Prior multisystemic therapy trials

The two promising MST-PSB trials [18, 32] were conducted in the United States by the developers of the intervention, and there are potential differences in recruitment, sample and the comparator condition when these trials are considered in relation to the UK context. The first efficacy trial [18] recruited participants exclusively from the juvenile justice system who had been adjudicated for a sexual offence, and the sample was also characterized by high rates of non-sexual offending. All participants in the comparison condition received non-manual-driven group cognitive-behavioural therapy (CBT). Although CBT does not have a well-developed evidence base for juvenile sexual offenders, it has proved efficacious when applied to several child mental health conditions such as anxiety [36, 37] and posttraumatic stress disorder [38]. CBT is also the preferred treatment approach for treating adult offenders (see [20]). By contrast, while the larger community-based MST-PSB effectiveness trial in the United States [32] also recruited participants solely from the juvenile courts system, the TAU was sex-offender-specific group treatment provided by probation, with a minority of participants accessing private mental health treatment.

In the United Kingdom, juvenile sex offenders are identified and receive mental health support and treatment through youth offending services and social care, where they are identified as being high risk, and in need of intervention and potentially out-of-home placement for safety reasons. Given the different ways in which young people displaying sexually harmful behaviour are referred in the United Kingdom, it is possible that participants in the UK trial will differ from their counterparts in the United States in the nature and severity of their problem sexual behaviour, as well as their co-occurring mental health disorders and problems. For example, mental health systems for identifying and treating young people showing problematic sexual behaviours are underdeveloped [39], and therefore, it is possible that young people showing both problematic sexual behaviours and conduct problems may not be identified to the same degree as those in the United States. The interventions provided to these young people are also specific to the UK mental health, juvenile justice and social care systems (see ‘Management as usual’, below). Finally, and more broadly speaking, the two previous MST-PSB trials have collected secondary outcomes at one time point, immediately following treatment [18] or up to 1 year following the interventions [32]. In order to understand better the longer term individual, family and school adjustment of participants following intervention, data in these domains need to be collected at more frequent intervals and over a longer time period to determine the utility of MST-PSB in the United Kingdom.

### The current trial

This paper describes the protocol for the Services for Teens Engaging in Problem Sexual Behaviour (STEPS-B) trial, a UK feasibility evaluation of MST-PSB. The MST-PSB intervention uses the term ‘problem sexual behaviour’ to describe young people who display serious non-normative sexual behaviours that victimize others, which can be aggressive or non-aggressive sexual acts [40]. Gerardin and Thibaut [41] defined this behaviour as committing any sexual act with a person of any age against the victim’s will, or in an aggressive, exploitative or threatening manner, including non-contact sexual acts such as exposing one’s genitals or masturbating in public.

The STEPS-B trial aims to assess the feasibility of implementing MST-PSB and also the barriers to implementing it: young people who display problem sexual behaviour suffer intense stigma and blame [42]. There are also well-documented challenges to effectively identifying, assessing and intervening with this clinical population in the United Kingdom [43]. As well as evaluating the effectiveness of MST-PSB in a UK context, the aim of the trial is to determine whether MST-PSB can be implemented fully and at a scale that would warrant a full trial. This feasibility trial will follow rigorous RCT guidelines, and therefore, part of the evaluation will be to determine whether a stringent RCT is realisable, as there have been no previous RCTs of interventions addressing adolescent problem sexual behaviour in the United Kingdom.

The MST-PSB feasibility trial is being conducted at the Brandon Centre, London, a charitable organization serving the mental health needs of young people. The Brandon Centre is participating in the RCT as part of its funding agreement with the UK Department of Health for having a clinical team. In the run-up to the trial, clinicians at the Brandon Centre will have been working with MST-PSB families for at least 18 months. This represents a bedding-in period to help raise awareness of the Centre’s specialized service among referrers (youth offending, social care and child and adolescent mental health services [CAMHS]), as well as ensuring that the therapists are applying the therapeutic model of MST-PSB (see below) in accordance with MST principles and guidelines. The Brandon Centre developed expertise in delivering MST for general antisocial behaviour (‘standard’ MST) in the first RCT of MST in the United Kingdom [25] and has continued to provide standard MST in several localities in London since then. While the MST-PSB clinical service is provided solely by one team of trained therapists at the Brandon Centre, recruitment for the MST-PSB trial is occurring across five localities in London.

There are specific feasibility issues in implementing an RCT for problem sexual behaviour in the United Kingdom. These include the challenge of recruiting an adequate sample of adolescents displaying sexually problematic behaviour; the acceptability of an RCT to the key agencies and systems that are involved in managing these young people and in providing them with mental health services; the ability to identify comparable interventions specifically designed to target problem sexual behaviour (that is, MAU); and the acceptability of the research protocol to young people and their families, given the stigma associated with individuals who display problem sexual behaviour and high levels of anxiety within families and professional systems regarding the management and treatment of this behaviour.

While the social and fiscal costs of adolescent sexual offending are substantive and warrant the development of effective and evidence-based services, European epidemiological studies of adolescent sexual offending in Oxfordshire, UK [44] and Sweden [45] suggest that there are low base rates of sexual offending and referrals to social services, respectively. The results of the Oxfordshire survey of sexual offending by males 12 to 17 years of age, using a postal questionnaire, revealed a 1-year incidence of 1.5 official juvenile sexual offenders per 1000 males (0.85 per 1000 12- to 17-year-olds of both sexes) [44], whereas the Swedish national survey of all adolescent sex offenders 12 to 17 years of age referred to social services in the year 2000 yielded a 1-year incidence of 0.060 % (95 % confidence interval = 0.052-0.068), or six referrals per 10,000 adolescents [45]. At the same time, sexual offending rates based on adolescent self-report are likely to underestimate the incidence of sexual offending in this population given that this behaviour is illegal and associated with shame and stigma. This assertion is supported by estimates of adolescent sexual offending based on reports of victims of sexual abuse. In addition, referrals to social services by professionals may be influenced by their perceptions of whether effective services will be available once a referral is processed.

The available data on prevalence and the difficulty gaining accurate estimates of incidence and service use suggest that the size of the pool of available participants to recruit into an RCT for problem sexual behaviour is uncertain. Therefore, the success of the trial is likely to depend on efficient identification of potential participants. It will also rely on established systems for providing specialist treatments for adolescent sexual offenders and for managing their mental health needs, given that judicial processes can determine the offenders’ availability for mental health interventions or delay timely provision of treatment. Two recent reviews of the provisions for juvenile sexual offenders in the United Kingdom noted that, despite examples of good practice, services for identifying and intervening with young people displaying problem sexual behaviour are underdeveloped [39]. For example, a recent examination of multi-agency responses to children and young people who sexually offend concluded that identification of young people’s problem sexual behaviour was often subject to disbelief, minimization and denial by professionals and by families; that much work was characterized by poor communication between the relevant agencies, with inadequate assessment and joint planning; and that cases were slow to get to court, on average taking 8 months between disclosure and sentencing, resulting in lengthy periods when little or no work was done with the young person [43]. The findings from this joint government inspection suggest that implementation of the MST-PSB trial will require substantial liaison and problem-solving with all relevant stakeholders to help identify young people who are appropriate for the trial and would benefit from the available interventions, as well as planning to accommodate judicial processes that will impact on the timely provision of both MST-PSB and MAU interventions.

Finally, the feasibility of the MST-PSB trial will undoubtedly depend on the acceptability of the research protocol to young people and their families. The research protocol for the MST-PSB trial, including the majority of the measures that will be administered to young people and their carers, has been successfully implemented with a diverse and large sample of young people showing antisocial behaviour and their carers in the START trial of standard MST [28]. However, the process of obtaining informed consent in the MST-PSB trial will depend on (1) a discussion between the young person and their carer(s) with an MST supervisor and a senior research assessor (RA), (2) details of the young person’s problem sexual behaviour, and (3) the completion of questionnaires by the young person and their carer(s) that involve details of their sexual development, including potential atypical sexual interests. It is possible that the young person and their carer(s) may be hesitant to participate in this process, given that the subject matter is highly sensitive, stigmatizing and often unknown to the wider community, and that adolescent problem sexual behaviour engenders high levels of anxiety in families and professional systems [39]. It is likely that effective relationships with the MST-PSB team, referral agencies, young people and their families will be paramount for the trial to be successful.

There are also several unresolved issues related to transporting MST-PSB to health and social care systems and jurisdictions outside the United States. In relation to a UK feasibility trial, the following issues require systematic monitoring: the clinical competence of therapists to practise MST-PSB in light of the curriculum differences for psychologists and social workers in the United Kingdom and the United States; the absence of data concerning the evidence base of usual services (that is, MAU) in the United Kingdom, given there are no prior RCTs of interventions for problem sexual behaviour in this country; differences in the motivation of MST therapists and the research team who were not involved the development of MST-PSB; and contextual issues such as differences in national standards with regard to sentencing policy and practice. In addition, while the sample sizes of studies with sexual offenders in general, and RCTs in particular, are relatively small, the larger MST-PSB effectiveness trial [32] provided no information regarding sample heterogeneity on key variables such as the presence or absence of non-sexual conduct problems [6] or the age differential between perpetrator and victim at randomization [10], both of which may influence the response to treatment.

### Aims

The aim of STEPS-B is to carry out a feasibility trial that will inform policymakers, commissioners of services and professionals about the potential for implementing MST-PSB as a programme for intervening with young people showing problem sexual behaviour. Should MST-PSB show potential in the United Kingdom context, the trial will determine what would be necessary to implement the programme more fully and at a scale that would warrant a full trial.

The trial will investigate whether MST is associated with the elimination or reduction of levels of sexual and nonsexual offending; reduction or elimination of problem sexual behaviours; reduction in nonsexual antisocial behaviour; less time spent in custodial institutions; and improved educational outcomes. It will also aim to establish the cost of MST-PSB relative to MAU, and the cost-effectiveness of providing this intensive form of intervention against the background of costs incurred in the 20-month period following randomization.

Finally, the trial will investigate specific feasibility issues that pertain to effective implementation of MST-PSB in the United Kingdom. These include: specific characteristics of young people’s problem sexual behaviour, where they are referred from, and what services have been or are being provided to them whether or not they are randomized into the trial; the beliefs and attitudes held by professionals around family intervention for this population, given that the young person will remain in the family home despite having committed sexually harmful behaviour towards other young people, including, in some cases, a sibling living in the same home; the degree to which professionals are supportive of the procedures and demands associated with an RCT research evaluation, including the randomization and its consequences; and the degree to which the institutions and agencies that manage young people showing problem sexual behaviour, such as the courts and child protection services, are able to support an RCT evaluation. It will also investigate the acceptability of the trial protocol to young people and their carers, and will gather information on their experience of MST-PSB and MAU interventions, through qualitative interviews following completion of treatment.

## Methods/Design

### Trial design

The STEPS-B trial is a single-site feasibility trial comparing MST-PSB with carefully documented MAU for adolescents who meet criteria for being at ‘high risk’ of requiring out-of-home placement, specifically when this risk is associated with problem sexual behaviour.

### Ethics

The study protocol was approved by the London-South East Research Ethics Committee (reference number 11/LO/0772). Research and development approval has also been sought and given for the Brandon Centre by Camden and Islington Research Ethics Committee (11/LO/1707).

### Study setting

The study will be conducted by the research team based at University College London (UCL). The team will be responsible for collecting data from cases referred to the Brandon Centre, London. Eighty families will be recruited to the study through referrals made to the Brandon Centre from at least five London boroughs. Referrals are anticipated from social care, CAMHS and youth offending services.

Participants will be recruited over 3 years. Recruitment will commence about 18 months after the therapists at the Brandon Centre have started working on MST-PSB cases; this initial period will allow MST-PSB therapists sufficient time to reach fidelity to the MST-PSB model before randomization begins. The team of three MST-PSB therapists is guided by a supervisor - a doctoral-level (or master’s-level with significant clinical and supervisory experience) therapist with sound knowledge of the theories and therapeutic practices underpinning MST-PSB and experience in providing MST clinical supervision. The MST-PSB supervisor conducts weekly group supervision, and one-to-one supervision of the MST-PSB therapists as needed, to ensure therapists are adhering to MST-PSB principles in their delivery of the intervention.

### Participants

Eighty participants will be recruited and randomly allocated, with half of the consecutive qualifying cases being assigned to MST-PSB and the other half to MAU.

### Sample size and power calculation

It is estimated that the Brandon Centre will have approximately two eligible cases per month, where referrals are obtained from a minimum of four boroughs, which would result in 72 cases randomized into the trial over the 3-year recruitment period. Five more boroughs are set to join the trial, resulting in nine boroughs in total; it is predicted that this will result in 34 eligible referrals per year, to give a total of approximately 102 cases randomized over 3 years.

**Fig. 1:**
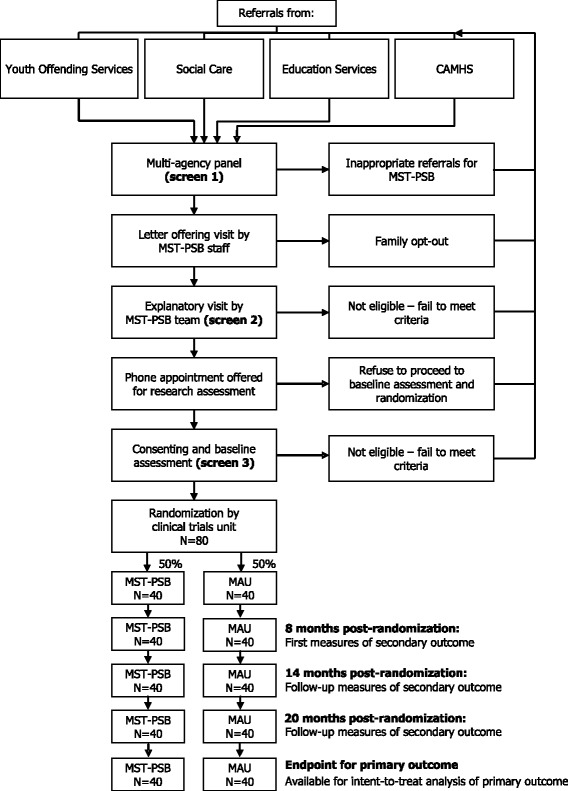
CONSORT flow diagram of progress through the phases of recruitment and treatment in the STEPS-B trial of multisystemic therapy for youth with problem sexual behaviour

Effect size has been estimated on the basis of the available data from a recent MST intervention study of sexual offenders in the United States [46]. As only summary statistics were available, 95 % confidence intervals for effect size were used and the lower limit chosen in a conservative approach. On the basis of the number of participants in the two groups who moved from offender to non-offender status during the follow-up period, the highly significant effect of treatment (*P* < 0.001, Fisher’s exact test) is 0.32 to 0.49 (Cramer, a medium to large effect). In the current study, non-offender status is defined as no arrest leading to conviction over the follow-up period, based on police computer records. We expect to achieve the lower end of the effect size range, given that the trial is mounted independently (although under the supervision) of the development team and involves the transportation of the clinical methodology across national boundaries, healthcare systems and cultures. A sample size estimate to give 80 % power based on the lower estimated effect size of 0.32 suggests 56 participants in a two-group design. Figure 1 shows the expected flow of participants from recruitment through to the end of the study.

### Eligibility criteria

Participants will be adolescents who meet the criteria for ‘high risk’ of requiring out-of-home care, specifically when this risk is associated with problem sexual behaviour.

#### Inclusion criteria

Inclusion criteria are as described below:

- Young people aged 10 to 17 years old
- Sufficient family involvement for MST-PSB to be applied: either living in the parental home, in long-term foster care, or in short-term out-of-home placement but family reunification is imminent (within 30 to 60 days)
- The young person and family are not already engaged in a problem sexual behaviour-specific treatment
- Problem sexual behaviour within the past year
- Risk of custody or out-of-home placement
- Risk of reoffending
- Displaying aggression at home, at school or in the community.

#### Exclusion criteria

Exclusion criteria are as follows:

- Young person is living independently or a primary caregiver cannot be identified
- The sexual offence is under police investigation at the time of referral
- The primary reason for referral is related to suicidal, homicidal or psychotic behaviours
- Caregiver persistently denies that the young person has engaged in problematic sexual behaviour
- Young person has pervasive developmental delays
- Young person has generalized learning problems (clinical diagnosis) as indicated by IQ <65
- Young person has severe substance misuse as the primary presenting problem.

### Recruitment and baseline procedures

The authors have developed a multiple gating procedure for recruitment, based partly on the experience of the largest adolescent mental health trial to date evaluating standard MST for youth displaying conduct problems [28], which, similarly, recruited complex families with multiple needs. Using this procedure, decisions about eligibility are made at three points: first, by a telephone discussion between the referrer and the MST-PSB supervisor; second, through discussions between the MST supervisor and the research team, based on information gathered from the referral form; and third, through discussions between the MST supervisor and the research team after having received additional information from an introductory home visit attended by the MST-PSB supervisor and an RA, with the purpose of explaining the rationale for the research trial and answering any questions about the MST-PSB and MAU services potentially available to the family.

In order to maximize recruitment and to ensure that our planned sample size is achieved, research personnel are involved in several activities with different parts of the system that may influence recruitment. Specifically, a member of the research team attends service team meetings and multi-agency panels that review potential referrals in the different boroughs, and there is regular contact with service leads and managers, which will influence the visibility and importance of the trial within services. Finally, ongoing service and agency awareness is complemented by ongoing presentations (for example, to youth offending evidence-based practice and judicial forums), a quarterly newsletter sent to all potential referrers, and an active website, which provides information for service teams about how to make a referral, a referral leaflet, a description of MST-PSB and the STEPS-B trial, and a ‘frequently asked questions’ section for young people, parents/carers and professionals.

#### Screen 1

Referrals are anticipated from youth offending services, CAMHS, social care and education services (see Fig. 1). Prior to the clinical trial, the MST-PSB team established close working relationships with the four referring boroughs through presentations, screening referrals, working with families and developing relationships with other service providers for youth displaying problem sexual behaviour.

Before a referral form is submitted, the potential referrer will complete a telephone case consultation with the MST-PSB supervisor. As the cohort is not homogeneous, individual cases will often require different levels of support. During the telephone consultation, the MST-PSB supervisor will screen the case against the eligibility criteria and begin to plan in parallel with the referrer another potential sexually harmful behaviour intervention, if the case and family are not randomly allocated to MST-PSB.

#### Screen 2

Once a referral has been accepted, the clinical team sends a standard letter to the parents (and separately to the young person if aged 16 or 17), informing them of the trial. In addition, the MST-PSB supervisor contacts the family by telephone to discuss the trial and arrange an introductory meeting.

The introductory meeting takes place at the family home, with the MST-PSB supervisor, senior RA, young person and primary carer(s) present. At this meeting, the nature of and concerns related to the young person’s problem sexual behaviour are reviewed and a credible MAU path (the best alternative treatment at that locality; see below), should the young person not be randomized to receive MST-PSB, is agreed upon by the young person, primary carer(s) and the trial team. Further information is also gathered regarding eligibility, such as agency involvement incompatible with participation in the trial (for example, the family is already engaged in a treatment that would interfere with MST-PSB), potential risk of harm to workers, or whether the young person has generalized learning problems. In the event that the primary carer steadfastly denies that the young person has engaged in problem sexual behaviour, the MST-PSB supervisor will offer a maximum of three sessions to explore the allegations with the primary carer(s), to see whether their position shifts. At the end of the introductory visit, the young person and primary carer(s) are given specifically tailored information outlining the trial, in age-appropriate and culturally appropriate language. The family is given contact details for the RA so that they can contact the RA if they have any further questions.

#### Screen 3 and consent

Unless the family expresses a decision not to participate at the time of the introductory visit, they are given a minimum of 3 days to consider whether they would like to join the clinical trial. If the RA does not hear from the family within the agreed time period, the RA telephones the family, answers any additional questions they may have, and arranges an appointment for a research assessment.

At the research assessment, each young person and primary carer(s) sign informed consent forms agreeing to join the clinical trial, including permission to access the police, correctional and educational databases, which remains in effect for 20 months. For families who sign the consent forms, the RA administers pre-randomization questionnaires and measures are completed during this contact (that is, before group assignment).

Once the young person and family’s eligibility have been confirmed and the instruments completed, the RA contacts the trial centre and relays the family’s randomization details. The clinical team informs the family and referrer of the outcome of randomization within 48 hours of the consent and pre-randomization visit being completed.

Once the family has completed the compulsory instruments to be able to join the trial, the young person is asked whether they would like to take part in the optional Child Attachment Interview (see Measures, below). If the young person and primary carer(s) agree, an additional meeting is arranged within 1 week of the date of randomization. At this additional visit, consent forms are signed by the primary carer(s) and young person and the Child Attachment Interview is conducted.

### Randomization and procedures to minimize bias

Following consent and the baseline (pre-randomization) assessment, a trial identification number is assigned to the participant. Eligible consenting participants are randomized to MST-PSB or MAU on a 1:1 basis by the Trials Unit at UCL through the use of a secure randomization service that ensures allocation concealment. A computer-generated adaptive minimization algorithm that incorporates a random element is used with the following stratification factors: gender, age (10–14, 15–17), conduct problems presenting with problem sexual behaviour (yes or no) and age differential between the perpetrator and victim (<4 years or ≥4 years). These strata were selected because previous research has shown that a greater age differential between perpetrator and victim, and whether the young person is also displaying antisocial behaviour, are associated with more severe maladjustment and poorer long-term outcomes [6, 47, 48].

To minimize the possibility that RAs will learn of the treatment allocation for any individual participant, the RAs are blind to treatment allocation at randomization, and there is no direct communication between RAs and therapists. This procedure has been used in the START trial of standard MST [28] and has proven to be effective.

### Planned interventions

#### Multisystemic Therapy for Problem Sexual Behaviour

MST is based on the premise that behaviour is multi-causal; therefore, to change an individual’s behaviour successfully, multiple drivers need to be addressed. Consequently, the approach works with the young person’s ecology (namely, the young person’s primary relationships, behaviour and the developmental challenges at home, school and in the community), as well as the young person themself; places a high premium on approaching each young person/family as unique; and has a specific focus on parental/carer involvement in treatment delivery, due to carer(s) being viewed as the key agents of change. MST has a track record of working with hard-to-reach, complex families with multiple needs. To increase engagement and formulation, a family is allocated one therapist who employs interventions that are strongly supported and informed by research. The sessions take place in the family home, and outreach work is undertaken when necessary. The family also has access to 24-hour/7 days a week telephone support with a therapist who is fully briefed on the family’s difficulties, safety plans and stage of therapy, so that support is readily available in times of crisis.

MST was originally developed to work with youth who engage in antisocial behaviour. In recent years, the research and implementation base of standard MST has been built upon to produce interventions to treat young people with a range of other difficulties, such as substance abuse, diabetes management, psychiatric care, child abuse and neglect, as well as problem sexual behaviour.

Problem sexual behaviour is an exclusion criterion for standard MST. MST-PSB, the adaptation of standard MST specifically for working with youths showing PSB, aims to reduce the level of both sexual and non-sexual offending in the target population. Supplementary to MST, MST-PSB has a greater focus on safety planning, individual factors (for example, impulsivity, social anxiety), and interventions specific to problem sexual behaviour, such as offence clarification sessions aimed at increasing accountability and safety and the promotion of normative sexual behaviour. Furthermore, family therapy techniques, such as structural and strategic family therapy interventions, are utilized to a greater extent than in standard MST, as well as peer interaction skills. In addition, where this is a factor, the impact of the young person’s own victimization and experience of abuse is assessed.

The intervention procedure consists of regular visits to the family home to meet with the young person and/or parent(s) for approximately 1 hour. Visits take place approximately three times per week at the beginning of the intervention and the frequency is reduced as the intervention progresses. Overall, the intervention time is 5 to 7 months.

In the STEPS-B trial, MST-PSB will be delivered by a team of at least three specially trained clinicians under the supervision of an MST supervisor, with weekly 1-hour conference calls for consultation with an MST Services staff member. It is expected that MST-PSB therapists will also have the support of consultation with local mental health professionals. To further support the therapists’ supervision and delivery of the intervention, a Therapist Adherence Measure-Revised (TAM-R) will be completed by the participants’ carers with an independent researcher every 3 weeks to review the therapist’s adherence to the nine principles of the MST model [49]. In addition, therapists will complete a Supervisor Adherence Measure bimonthly to rate the supervisor’s adherence to the nine principles.

The MST-PSB team at the Brandon Centre is centrally funded by the Department of Health and will have been operational for 18 months by the start of the clinical trial; during this period the intervention has been offered to referrers free of charge (otherwise, cost is approximately £10,000 per case). This bedding-in period has established the team in the referring boroughs, which has led to relationships being built with referring agencies and other intervention services addressing harmful sexual behaviour and to referrers increasing their awareness and knowledge of the referral criteria and intervention. Furthermore, as the MST-PSB team has been funded on condition that it participates in the research trial, greater collaboration is achieved between the MST-PSB team and research team by working together to meet joint targets such as recruitment numbers. Finally, the initial 18-month period will have given the MST-PSB team experience of implementing and addressing challenges to delivering the intervention in a UK context, therefore increasing treatment fidelity prior to the start of the transportability evaluation. To further ensure treatment fidelity, the MST developers have developed an extensive quality assurance system [50] and, in common with all MST sites, the Brandon Centre is licensed by MST Services (Charleston, SC, USA).

#### Management as usual

MAU constitutes the way in which a case would be managed if MST-PSB was not available. The authors aim to compare MST-PSB with specialist interventions for adolescents displaying problem sexual behaviour provided through social services, youth offending services and CAMHS. It is not assumed that MAU will be uniform across the referring boroughs. Instead, it is expected that the length, intensity and models of intervention will vary. Furthermore, it is envisaged that some young people assigned to MAU may not receive any interventions, owing to the limited service provision available where they live. The trial will map the specific profiles of services delivered to youth randomized to the MAU arm of the trial, as well as the approaches used within MST-PSB.

The service to which a young person may be referred for MAU is likely to depend on the severity of the case, the interventions available in the borough’s directorate and the provision for the borough to spot purchase. Potential options for MAU may include: AIM (Assessment Intervention and Moving on), an assessment and intervention programme that can be delivered by CAMHS, youth offending teams and social care practitioners; Youth In Need, a group CBT programme with an emphasis on the developmental perspectives underpinning offence-related issues; The Portman Clinic, which provides individual therapy using a psychoanalytical model; SWAAY Child and Adolescent Services Ltd or the Bracton Centre, both of which offer residential placements for young people where they can access a variety of individual and group programmes specifically targeting problem sexual behaviour as well as attending to the young person’s educational needs; and practitioners in CAMHS, social care and youth offending services who have developed expertise in working with this client group. Depending on the seriousness of the problem sexual behaviour, the young person may attend the NSPCC’s National Clinical Assessment and Treatment Service (the NSPCC is a UK children’s charity with a major focus on abuse prevention and intervention), which offers assessment, a multidisciplinary risk assessment report and treatment. The project’s intervention models include CBT, psychotherapy, individual psychological therapy and group therapy.

### Assessments and outcome measures

To maximize the clinical validity of the outcome evaluations, assessments are being made using multiple methods (for example, objective offending indices, semi-structured interviews, standardized questionnaires) completed by different informants (for example, the young person, carer(s) and teacher(s)) who are significant in the multiple domains that characterize young people’s functioning (home, school, community).

#### Feasibility outcomes

The feasibility outcomes measure chosen variables that the investigators hypothesize will be related to the effective implementation of MST-PSB in the United Kingdom.

First, it is predicted that specific characteristics of young people referred for displaying problematic sexual behaviour will be more likely to result in these young people being randomized into the trial. The specific characteristics that will be examined include the age of the young person, severity of the young person’s problematic sexual behaviour, source of the referral, and whether or not the young person was involved in judicial proceedings at the time of randomization.

Second, the beliefs and attitudes held by professionals working in the agencies and services that will refer into the trial will be evaluated. The data gathered concerning their beliefs and attitudes will include the degree to which they are supportive of a family-based intervention for a young person displaying problematic sexual behaviour; the degree to which they are in accord with the procedures and demands associated with an RCT research evaluation, including the randomization and its consequences; and the degree to which they view the institutions and agencies that manage young people showing problematic sexual behaviour as able to support an RCT evaluation.

The trial team has developed a bespoke 15-item questionnaire (the Feasibility Questionnaire) to assess beliefs and attitudes in the following areas: family treatment as an appropriate intervention for young people showing problematic sexual behaviour (for example, ‘My preference would be for 1:1 work to take place with a young person who displayed problematic sexual behaviour over a family approach’); support for RCT evaluations of young people showing problematic sexual behaviour (for example, ‘I have ethical concerns when referring a young person into a clinical trial where the young person is randomly assigned to an intensive treatment or a control group (which may involve a less intensive intervention)’); and perceptions of the systemic context (agencies and institutions) as facilitative of RCT evaluations (for example, ‘I am knowledgeable of different services that support young people who are perpetrators of problematic sexual behaviour (excluding MST-PSB)’). These three areas have been chosen on the basis of preliminary discussions with referrers and agencies that manage young people showing problematic sexual behaviour in the localities where recruitment will take place, as well as our previous experience liaising with referrers and the major agencies involved in providing CAMHS while conducting two earlier RCTs of standard MST, including a large multi-site trial [28]. The experience of implementing previous RCTs has been considered in light of the guidelines for strategic arrangements, assessment and planning, and interventions set out by the joint inspection of the effectiveness of multi-agency work with children and young people who have committed sexual offences in the United Kingdom [43]. The Feasibility Questionnaire will be completed by approximately 100 practitioners who are involved in the management and treatment of young people who show problematic sexual behaviour and who will potentially refer participants to the trial.

Third, the acceptability of the trial protocol to young people and their carers will be investigated through qualitative interviews following the completion of treatment. The qualitative interviews concerning participants’ experience and views of the trial protocol will be part of a larger semi-structured interview, which will include discussion of their experiences of the MST-PSB and MAU interventions (see ‘Interviews’ below). The part of the interview that elicits information regarding the trial protocol will focus on several domains: the process of identifying and discussing the young person’s problematic sexual behaviour as part of the family visit at which consent is obtained; the explanation of randomization and their understanding of the two treatment options available to them; and their perceptions regarding the appropriateness and challenges of completing the study’s standardized measures.

#### Primary outcomes

The primary outcome is the proportion of documented cases of offending within 20 months of randomization, based on police records supplemented by information obtained from the young person, parents/caregivers and social care services. While this may be only a partial or even an inaccurate reflection of the psychological conditions and social behaviours of the participants, it is the indicator of primary concern for the funders of the programme as, in most cases, this record triggers costly juvenile justice and social interventions. A linked outcome, of similarly high concern to funders of services, is assignment to long-term (3 months or longer) out-of-home placements in specialist residential provision when social services consider that the safety of the participant’s family or community is compromised by the individual, even in the absence of arrest and conviction. This means all participants who are placed into local authority care, are incarcerated, long-term hospitalized or offered residential schooling at any time in the 20 months following randomization will not be considered to have moved from the offending to the non-offending group. This information will be obtained from caregivers and cross-referenced with documented information from the national social care services database to maximize accuracy. The investigators expect this trial to give information on how many young people assigned to MAU and to MST-PSB require specialist residential provision either immediately after intervention or during the follow-up period.

#### Secondary outcomes

Secondary outcomes are limited in number to reduce the measurement burden on young people and their primary carer(s), as the investigators’ previous experience has shown that having too many measures to complete is a disincentive to continued participation [25, 28]. The domains that the investigators consider key to the intervention, and consistent with previous RCTs of MST-PSB, are youth sexual and non-sexual offending, adolescent well-being and family functioning. Although rarely measured to date, we will also assess deviant sexual interests, given their specific association with adolescent sexual offending found in a recent meta-analysis seeking to understand what differentiates adolescent sexual offenders from general antisocial offenders [10]. Economic data that are highly relevant to conducting a comprehensive cost-benefit analysis will be collected alongside these outcomes. The study will also collect data on variables associated with key mechanisms of change that are documented in the literature on standard MST [49] and confirmed in trials of sexual offenders [46]: parent-adolescent relationships and young people’s associations with deviant peers. Furthermore, the study will evaluate parenting skills in detail, given that the MST model aims to improve outcomes for young people by targeting caregivers as being primarily responsible for facilitating change. Adolescent symptoms have been shown to decrease with increased supportiveness and decreased conflict between parents [51, 52], and decreases in youth antisocial behaviour, deviant sexual interests and sexual risk behaviours have been associated with caregivers’ ability to follow through with disciplinary practices and having decreased concern about their adolescents continuing to associate with antisocial peers [46]. Furthermore, adherence to the MST manual by therapists appears to improve treatment outcomes: treatment fidelity leads to better family functioning, which in turn decreases deviant peer affiliation, leading to decreased delinquent behaviour [49].

#### Objective measures

Objective outcomes will be collected from reports of sexual and non-sexual offending behaviour based on police computer records, including details of custodial sentences. These measures will be taken at 6-month intervals, for the 6 months before randomization, the 8 months covering the intervention period (adjusted as MST-PSB lasts on average 5 to 7 months), and 6-monthly until the 20-month follow-up point. The number of records of offending behaviour (count data) will be obtained and 6-month periods free of any offending behaviour will also be recorded (binary data). Records will be obtained from the Police National Computer as well as from the Young Offender Information System database; these records detail information on offences, court appearances, criminal orders, police custody records and arrest rates. Records of school attendance and exclusions will be retrieved from both the schools themselves and the National Pupil Database to assess educational outcomes.

#### Self-report

The RA will administer pre-testing questionnaires during the initial contact with the young person and family after they have consented and before group assignment. Post-testing by the RA will take place 8 months after entry into the study; it is intended that this will be a minimum of 2 weeks after the family completes the intervention (MST-PSB or MAU). Follow-up assessments will be made at 14 and 20 months post-randomization. Self-report measures of well-being and adjustment, antisocial behaviour, inappropriate sexual interests and difficulties, parenting skills and family functioning, as well as parental mental health and adjustment, will be collected.

#### Well-being and adjustment

A general assessment of well-being will be obtained using the Strengths and Difficulties Questionnaire [53]. Depression will be monitored using the Short Mood and Feelings self-report questionnaire [54], completed by the adolescents. Educational outcomes will include evaluation of participants’ emotional and behavioural functioning in the classroom using the Conners’ Comprehensive Behaviour Rating Scale [55].

#### Sexual adjustment and difficulties

The Multidimensional Inventory of Development, Sex, and Aggression (MIDSA) [56] and the Adolescent Sexual Behaviour Inventory (ASBI) (parent and youth forms) [57] will be used to assess the young person’s sexual adjustment, with a focus on inappropriate sexual interests and difficulties, as well as any prior history of maltreatment.

#### Antisocial behaviour

The prevalence and incidence of delinquent behaviour (for example, vandalism, theft, or burglary) and of peer delinquency will be assessed using the Self-Report Delinquency measure [58]. Non-compliance and increasingly serious forms of antisocial behaviour, as well as young people’s perceptions of law-abiding behaviour and institutions, will be assessed using the Antisocial Beliefs and Attitudes Scale [59]. Callous and unemotional traits will be assessed using the Inventory of Callous-Unemotional Traits, an updated version of the Antisocial Process Screening Device [60]. The investigators predict that MST-PSB will achieve decreases in adolescents’ associations with antisocial peers, increases in positive peer relations and greater commitment to prosocial activities. This prediction is consistent with the MST model and hypothesized mediating mechanisms [46] and is relevant to social policy initiatives and concerns.

#### Parenting skills and family functioning

The quality of the relationship between adolescent and carer(s), family functioning and parenting practices will be assessed using the Family Adaptability and Cohesion Scales [61], the Inventory of Parent and Peer Attachment-Revised [62] and Loeber et al.'s parent-completed measure of positive parenting and disciplinary practices, along with parental monitoring and supervision [63] . Parental disruption and conflicts will be assessed using the short form of the Conflict Tactics Scale [64].

#### Parental mental health and adjustment

Parental mental health will be briefly assessed using the General Health Questionnaire-28 [65], a commonly used screening instrument.

### Interviews

#### Demographics interview

A bespoke interview (Demographic Interview for Parents) covering general family information, including parental forensic history, schooling and economic information, constructed by the investigators, will be administered to all parents.

#### Interviews involving young people

Child psychometrics will be obtained by using the Wechsler Abbreviated Scale of Intelligence [66]. Psychiatric disorders will be identified and a psychosis screen provided by the Development and Well-Being Assessment [67], a computerized structured interview measure that will be administered to the carers and young people. The quality of the parent-adolescent attachment relationship will be assessed with the Child Attachment Interview [68].

#### Experience of MST

After the intervention, young people and their carer(s) will be interviewed to elicit their views regarding their experience of MST and factors that they perceive had facilitated or inhibited therapeutic change, based on qualitative research carried out during the Brandon Centre trial [69] and the more recent START trial [28].

### Health economic evaluation

Health economic analysis will be conducted by the Centre for the Economics of Mental Health at the Institute of Psychiatry, London. This analysis will explore the relative costs and cost-effectiveness of MST-PSB and MAU. The evaluation will take a broad perspective and will include all health, social services, education and voluntary sector services, as well as costs to the criminal justice sector, costs resulting from crimes committed, and out-of-pocket expenses to the young people and their families.

Data on MST contacts will be collected directly from the Brandon Centre to avoid participants revealing their group allocation to the RAs. Data on the use of all other services will be collected in interviews using the Child and Adolescent Service Use Schedule (CA-SUS) and EQ-5D questionnaire, which have previously been used with young people with complex mental health and social care needs [70–73]. The CA-SUS will be adapted to the current study population by review of the literature and pilot testing, to ensure comprehensive coverage and face validity. The cost of the MST-PSB and MAU interventions will be calculated through a detailed micro-costing (or bottom-up) approach using standard costing methodology [74, 75]. This will involve estimation of the indirect time spent on individual cases, including preparation, meetings, telephone calls and supervision, as well as detailed recording of direct face-to-face contact.

### Programme components of Multisystemic Therapy for Youth with Problem Sexual Behaviours and management as usual

A bespoke fidelity measure, the Children and Young People - Resources, Evaluation and Systems Schedule (CYPRESS) (S. Pilling, S. Butler, C. Gaffney, P. Fonagy, personal communication), will be used to enable accurate characterization of the programme components of MST-PSB and MAU interventions. This approach is based on the initiative of the MST developers [50], who undertook a large-scale transportability study of standard MST involving families, therapists, supervisors and consultants. Organizational structure and climate as rated by the therapists, supervisors and consultants appeared to predict the outcome of MST [76]. CYPRESS has been designed to characterize care pathways for antisocial youths in the UK context. The measure will be administered in an interview format to service managers and therapists, to elicit care-pathway-relevant information in three main domains: ethos and service characteristics (for example, service has a comprehensive and shared view of the model of care provided; service has explicit criteria describing the population served); team operations (for example, service has a comprehensive model of supervision that provides for all team members); and range of interventions available to young people and their families (for example, a range of individual, group or family interventions are provided by the team, which are consistent with the model of care). The use of CYPRESS will allow key programme elements associated with outcome to be identified. The use of a common measure will allow a comparison between MST-PSB and MAU in the three domains described above and potentially provide information on common aspects of service function associated with outcome. This type of approach has been used to characterize other complex interventions in mental health [77]. It will be of particular value in assessing the key beneficial organizational components of such a complex intervention as MST-PSB when it is deployed in a healthcare system radically different from that in which it was developed.

### Qualitative interviews

To obtain additional information about participants’ experience of MST-PSB and being in an RCT of this treatment, the semi-structured interview developed by Tighe and colleagues [69] in the Brandon Centre trial will be adapted for young people showing problem sexual behaviour. This 45-minute interview will elicit carers’ and young people’s experience of having participated in an RCT for problem sexual behaviour (that is, their experiences of discussing their harmful sexual behaviour as part of the initial visits prior to consent, completing the measures, and treatment allocation), their experiences of having received MST-PSB or MAU services, and their views of the costs and the benefits. Semi-structured interviews will be conducted to explore how young people and their parents or carers experienced MST-PSB or MAU, whether they think that the young person’s functioning or carers’ lives have changed following MST-PSB (in areas targeted by the intervention) or MAU, and whether family relationships have changed. If the young person or carer reports changes, the interview will also explore the timing of those changes and how MST-PSB or MAU affected the changes. Interviews will be audio recorded and transcribed verbatim. The interviews may broach sensitive and stigmatizing issues with young people and their carers pertaining to their having received treatment for sexually abusive behaviour. To the investigators’ knowledge, young sexual offenders are rarely provided with the opportunity to reflect on their difficulties, treatment and social attitudes toward their behaviour. Thus, the interviews may provide valuable insights that will inform treatment delivery.

Transcripts will be analysed thematically using Interpretative Phenomenological Analysis (IPA). The primary objective of IPA research is to understand how individuals make sense of their experiences [78]. The intention is for the interview to be administered to approximately 12 to 14 % of families per treatment condition by an interviewer who is independent of the intervention but not blind to treatment allocation. Specifically, doctoral students who are trained and supervised by experienced qualitative researchers will conduct the interviews. The qualitative interviewers are separate from the MST-PSB service providers and the RAs. A random sample of the audio-taped interviews will be re-rated by independent raters to examine the reliability of qualitative coding.

### Follow-up assessment

Follow-up assessments will be conducted at 8, 14 and 20 months post-randomization. Table 1 shows a detailed outline of the planned measures at each follow-up point throughout the trial.

**Table 1:** Measures and schedule of administration in the STEPS-B trial of multisystemic therapy for problem sexual behaviour

Measure (abbreviation)	Parent		Young person		Independent source	Domain	Study month			
	Questionnaire	Interview	Questionnaire	Interview			0	8	14	20
Demographics Interview for Parents (DIP)		✓				Background	✓			
Social Service Records and Interview (SSR)		✓		✓	✓	Out-of-home placements		✓	✓	✓
Police Computer Records Report (PCRR)					✓	Youth antisocial	✓	✓	✓	✓
Exclusions from the National Pupil Database (NPD)					✓	Youth education	✓	✓	✓	✓
Strengths and Difficulties Questionnaire (SDQ)	✓		✓			Youth mental health/well-being	✓	✓	✓	✓
Conners’ Comprehensive Behaviour Rating Scale (CBRS)	✓					Youth school functioning; hyperactivity/impulsivity	✓	✓	✓	✓
Inventory of Callous-Unemotional Traits (ICU)	✓		✓			Youth callous-unemotional traits	✓	✓	✓	✓
Child and Adolescent Psychiatric Assessment (DAWBA)		✓		✓		Youth mental health profile	✓		✓	
Self-Report Delinquency (SRD)			✓			Youth antisocial	✓	✓	✓	✓
Adolescent Sexual Behaviour Inventory (ASBI)	✓		✓			Youth sex-related behaviours	✓	✓	✓	✓
Short Moods and Feelings Questionnaire (MFQ)			✓			Youth depression	✓	✓	✓	✓
Wechsler Abbreviated Scale of Intelligence (WASI)				✓		Youth psychometrics	✓			
Antisocial Beliefs and Attitudes Scale (ABAS)			✓			Youth antisocial cognition	✓	✓	✓	✓
Multidimensional Inventory of Development, Sex, and Aggression (MIDSA)			✓			Youth emotional denial and intimacy	✓	✓	✓	✓
The Inventory of Parent and Peer Attachment - Revised (IPPA-R)			✓			Youth parental attachment style	✓	✓	✓	✓
Child Attachment Interview (CAI)				✓		Youth attachment	✓		✓	
Loeber Caregiver Questionnaire (LCQ)	✓		✓			Quality of parenting	✓	✓	✓	✓
Family Adaptability and Cohesion Scales (FACES-IV)	✓					General family function	✓	✓	✓	✓
Child and Adolescent Service Use Schedule (CA-SUS)		✓		✓		Service use (economics)	✓	✓	✓	✓
Conflict Tactics Scales (CTS)	✓					Parental disruption	✓	✓	✓	✓
General Health Questionnaire-28 (GHQ-28)	✓					Parent mental health	✓	✓	✓	✓
EQ-5D			✓			Youth measure of health outcome	✓	✓	✓	✓
Children and Young People - Resources, Evaluation and system Schedule (CYPRESS)					✓	Service context				✓
Qualitative interview		✓		✓		Youth and parental experience of multisystemic therapy for youth with problem sexual behaviours (MST-PSB)		✓		

### Statistical analysis plan

All analysis will be according to the intention-to-treat principle. The characteristics of the treatment groups will be described at baseline. Preliminary analysis will investigate the pattern of missing follow-up data.

### Primary outcome

The primary outcome of the proportion of cases where a sexual offence has been incurred during the follow-up period will be described by a Kaplan-Meier graph and summarized by the proportions in each group with a new sexual offence by 20 months. The linked outcome of out-of-home placement will also be described by a Kaplan-Meier graph and summarized by the proportions in each group with out-of-home placement by 20 months. The number of events will be estimated in order to inform power calculations for a fully powered efficacy trial.

For time-to-event offending outcomes, the event rate will be estimated. For continuous secondary outcomes, such as questionnaire data, linear mixed effects models will be fitted in order to estimate the individual-level residual variance.

### Secondary outcomes

The feasibility outcome evaluating individual and systemic factors associated with recruitment into the trial will be analysed using logistic regression. The total score and subscale scores on the Feasibility Questionnaire will undergo descriptive analyses to characterize the direction and range of attitudes by referrers involved in the MST-PSB trial. As noted above, qualitative interviews eliciting the views of young people and their carer(s) on the underlying principles and demands of participating in an RCT will be analysed using IPA.

## Discussion

There is very little research that has evaluated therapeutic interventions for young people displaying problem sexual behaviour, and to date, no RCTs have been conducted in the United Kingdom. Although the results from two RCTs of MST-PSB [18, 32] have demonstrated the success of this intervention in reducing adolescent problem sexual behaviour in the United States, the evaluation of MST-PSB and adolescent problem sexual behaviour is still in its infancy. The STEPS-B trial aims to assess the feasibility of implementation of MST-PSB in the United Kingdom. The investigators hope that the feasibility trial will determine whether MST-PSB can be implemented fully and at a scale that would warrant a full trial. In addition, in following rigorous RCT guidelines, the trial will also assess whether the implementation of a stringent RCT evaluation of adolescent problem sexual behaviour is possible. Finally, this study will be the first RCT of MST-PSB that has not been conducted by the developers of the intervention. As such, it will provide a robust test of the effectiveness of this promising intervention as well as its transportability to outside the United States.

Evidence to support MST-PSB remains limited. Both prior evaluations of MST-PSB were conducted in the United States by the developers of the intervention and recruited participants solely through the juvenile court system. Moreover, in one of these two trials, measures of critical sexual and non-sexual reoffending outcomes were not reported and, as noted by the authors, the sample of young sexual offenders displayed very low levels of general psychopathology [32]. This finding appears to run contrary to knowledge that young people displaying problematic sexual behaviour have significant difficulties in other areas of their lives [8–10]. Consequently, while the evidence from the United States suggests that MST-PSB is a promising treatment when participants are recruited through the court system and when the intervention involves the developers of the programme, the question of whether MST-PSB is effective in reducing problem sexual behaviour in adolescents requires further research [17]. Furthermore, its potential applicability to the United Kingdom has yet to be tested. Moreover, the provision for adolescents who display problem sexual behaviour in the United Kingdom is difficult to compare with that in the previous evaluations, and the degree to which samples recruited into trials in the United Kingdom and the United States will be similar is unknown. It is clear that further high-quality RCTs with minimal involvement of the developers and long-term follow-up in the community are required to identify effective interventions for adolescent problem sexual behaviour and specifically to test the effectiveness of MST-PSB.

The primary aim of this study is therefore to conduct a rigorous, community-based trial in which MST-PSB is compared with the range of services that are typically provided to adolescent sexual offenders in the United Kingdom (that is, MAU). Whereas there is no good evidence that MAU for these young people and their families works, there is some evidence available to suggest that MST-PSB is of benefit. Therefore, this evaluation of both treatment options will enable closer observations of MAU services to provide an evaluation of interventions currently available in the localities, as well as MST-PSB. This pragmatic trial will inform policymakers, commissioners of services and professionals about the potential of MST-PSB in the UK context. Specifically, it will provide evidence to determine whether the provision of MST-PSB could reduce the incidence of out-of-home placements for young people at risk of being removed from their homes primarily because of problem sexual behaviour.

To ensure that the study achieves the fairest and least biased assessment of the potential benefits of MST-PSB, the investigators have designed the study with the support of MST Services but will carry out the RCT independently from the developers; however, the developers provide regular consultation to the clinical service at the Brandon Centre to ensure that MST-PSB is delivered to the highest levels of fidelity. As part of the feasibility evaluation, individual and systemic factors will be assessed that may be associated with young people being recruited into the trial, the beliefs and attitudes held by practitioners about the usefulness of empirically evaluating services for problematic sexual behaviour in the United Kingdom, and the perceptions of young people and their carers of having participated in an RCT for problematic sexual behaviour and of the intervention that they received.

The research is being conducted by a team based at UCL, with data collection from cases referred to the Brandon Centre. MST-PSB aims to reduce levels of both sexual and non-sexual offending in the target population, and so offending will be used as a key measure to determine whether the intervention is effective. In addition, as out-of-home placement (whether this constitutes incarceration, hospitalization, residential schooling or local authority care) represents an unhelpful outcome for antisocial adolescents in the majority of cases, the investigators have chosen to use preservation of the family (that is, the *absence* of out-of-home placement) as the main measure of potential benefit. Other possible benefits of MST-PSB will also be examined, such as the impact on the young person’s mental health and well-being, and that of the carer(s).

To address factors associated with improvement, the investigators have taken considerable effort to monitor the effect of MST-PSB on parenting and family functioning. It is anticipated that improvement observed in association with MST-PSB will be commensurate with the impact that the intervention has on variables within the family, and for other clinically critical outcomes (for example, antisocial peer group affiliation), to be in line with changes in the purported mediating processes [46].

To assess what the benefits of MST-PSB and MAU might be, care will be taken to gather accurate descriptions of the interventions delivered to both groups in the study. In addition, the investigators will attempt to chart the subjective experience of all stakeholders in the project (that is, participants, their carers and providers and commissioners of services). The data from the trial will be analysed to determine whether the expected benefit of MST-PSB is achieved and will provide initial data on whether MST-PSB represents an economically viable option for intervening with young people displaying problem sexual behaviour.

We anticipate that the trial will also have positive effects on practice with this group of young people, irrespective of the trial outcomes. As noted, there is a lack of coherence within and across the systems that manage these young people; there are challenges to effectively identifying, assessing and intervening with this clinical population; and there has been very limited development of evidence-based interventions [43]. As we develop relationships with the referrers and clinical services devoted to working with this population, we will help them to develop pathways for identifying and assessing problematic sexual behaviour in terms of criteria that are evidence-based, and will encourage them to identify credible interventions that respond to clearly defined needs and that will function as suitable comparators to MST-PSB. This feasibility trial, while specifically evaluating MST-PSB versus the MAU typically available for young people showing problematic sexual behaviour, is consistent with UK government policy that strongly advocates and funds evidence-based practice for children and young people [79, 79].

Finally, the qualitative aspects of the study, which will elicit the views of this stigmatized and marginalized group of young people, will provide valuable information about their lived experiences as sexual offenders, while the assessment of practitioners’ views specifically regarding provision of family and evidence-based treatments for this population will help us to understand the mental health context within which evidence-based services will need to develop. The qualitative data will complement the quantitative results about the treatment effectiveness of both MST-PSB and currently available services to enrich our understanding of the strengths and challenges facing mental health services for young sexual offenders in the United Kingdom.

## Trial status

The trial is in its third year and recruitment is ongoing.

## Abbreviations

CAMHS: Child and Adolescent Mental Health Services
CA-SUS: Child and Adolescent Service Use Schedule
CYPRESS: Children and Young People - Resources, Evaluation and Systems Schedule
MAU: Management as usual
MST: Multisystemic therapy
MST-PSB: Multisystemic Therapy for Youth with Problem Sexual Behaviours
RA: Research Assessor
RCT: Randomized controlled trial
TAM-R: Therapist Adherence Measure-Revised
